# Fracture Rate, Quality of Life and Back Pain in Patients with Osteoporosis Treated with Teriparatide: 24-Month Results from the Extended Forsteo Observational Study (ExFOS)

**DOI:** 10.1007/s00223-016-0143-5

**Published:** 2016-04-30

**Authors:** Bente L. Langdahl, Östen Ljunggren, Claude-Laurent Benhamou, Fernando Marin, George Kapetanos, Tomaz Kocjan, Eric Lespessailles, Nicola Napoli, Tatjana Nikolic, Helmut Petto, Thomas Moll, Erik Lindh

**Affiliations:** 1 Department of Endocrinology and Internal Medicine, Aarhus University Hospital, Tage Hansens Gade 2, 8000 Aarhus, Denmark; 2 Department of Medical Sciences, Uppsala University, Uppsala, Sweden; 3 Orléans Hospital, Orléans, France; 4 Eli Lilly and Company, Windlesham, Surrey, UK; 5 Papageorgiou General Hospital, Thessaloniki, Greece; 6 University Medical Centre, Ljubljana, Slovenia; 7 EA-4708-I3MTO, University of Orléans, Orléans, France; 8 University Campus Bio-Medico, Rome, Italy; 9 University Hospital, Zagreb, Croatia

**Keywords:** Osteoporosis, Observational study, Teriparatide, Fractures, Quality of life, Back pain

## Abstract

We describe the pre-planned interim analysis of fracture outcomes, health-related quality of life (HRQoL) and back pain in patients with severe osteoporosis treated with teriparatide for up to 24 months in the Extended Forsteo (Forsteo^®^ is a registered trade name of Eli Lilly and Company) Observational Study (ExFOS), a prospective, multinational, observational study. Data on incident clinical fractures, HRQoL (EQ-5D questionnaire) and back pain [100 mm visual analogue scale (VAS)] were collected. The number of patients with fractures was summarised in 6-month intervals and fracture rate over each 6-month period was assessed using logistic regression for repeated measures. Changes from baseline in EQ-5D and back pain VAS were analysed using mixed models for repeated measures. Of 1454 patients in the active treatment cohort, 90.6 % were female and 14.4 % were taking glucocorticoids. During teriparatide treatment (median duration 23.7 months), 103 patients (7.1 %) sustained a total of 122 incident clinical fractures (21 % vertebral, 79 % non-vertebral). A 49 % decrease in the odds of fractures and a 75 % decrease in the odds of clinical vertebral fractures were observed in the >18- to 24-month period versus the first 6-month period (both *p* < 0.05). EQ-5D scores and back pain VAS scores were significantly improved from baseline at each post-baseline observation during teriparatide treatment. In conclusion, patients with severe osteoporosis showed a significant reduction in the incident fracture rate during 24 months of teriparatide treatment in routine clinical practice, accompanied by a significant improvement in HRQoL and reduction in back pain. Results should be interpreted in the context of the non-controlled design of this observational study.

**Electronic supplementary material:**

The online version of this article (doi:10.1007/s00223-016-0143-5) contains supplementary material, which is available to authorized users.

## Introduction

Osteoporosis is characterised by low bone mineral density (BMD) and deterioration in bone quality resulting in increased bone fragility and predisposing patients to fracture. In 2010, the estimated number of people in the European Union (EU) with osteoporosis was 27.6 million and the annual number of new fragility fractures was 3.5 million; these included 610,000 hip fractures, 560,000 forearm fractures and 520,000 vertebral fractures [1]. Osteoporotic fractures, especially those of the hip and vertebrae, can cause pain and functional disability, reduce health-related quality of life (HRQoL) [2–6], and are associated with an increased mortality risk [7].

Teriparatide (recombinant human parathyroid hormone, Forsteo^®^ [8]) is an osteoanabolic agent that stimulates bone formation and improves bone quality and strength. Teriparatide was first approved by the European Medicines Agency (EMA) in June 2003 for up to 18 months of treatment in postmenopausal women with osteoporosis after a phase III randomised controlled trial (RCT) demonstrated that teriparatide reduces the risk of new vertebral and non-vertebral fractures in postmenopausal women with severe osteoporosis [9]. The reduction in the risk of new fractures seen in the RCT was confirmed in observational studies conducted in real-life settings, such as the European Forsteo^®^ Observational Study (EFOS), which showed a reduced incidence of clinical fractures, improved HRQoL and reduced back pain during up to 18 months of treatment with teriparatide in postmenopausal women with severe osteoporosis [10].

After the initial EMA authorisation, teriparatide received additional approval for the treatment of osteoporosis in men at increased risk of fracture and for the treatment of osteoporosis associated with sustained systemic glucocorticoid (GC) therapy in men and women at increased risk for fracture [11, 12]. More recently, the EMA has also approved teriparatide treatment for 24 months’ total duration [8]. In the USA, the observational Direct Assessment of Non-Vertebral Fractures in Community Experience (DANCE) study showed a reduction in non-vertebral fractures in men and women with osteoporosis treated with teriparatide for up to 24 months [13].

The Extended Forsteo^®^ Observational Study (ExFOS) is a non-interventional, prospective, observational study being conducted in Europe according to all approved indications and the extended treatment duration during the course of normal clinical practice. It also includes a post-treatment follow-up of at least 18 months [14] to assess post-teriparatide treatment patterns and effectiveness in normal clinical practice. The objectives of this pre-planned interim analysis of men and women with severe osteoporosis on active treatment with teriparatide for up to 24 months are to describe fracture outcomes, HRQoL, and back pain.

## Methods

### Study Design and Patients

Patients at 110 centres in eight European countries (Croatia, Denmark, France, Greece, Italy, Norway, Slovenia, and Sweden) were enrolled in ExFOS; the study design and baseline characteristics of the enrolled population have previously been described [14]. The study was designed with two phases: (1) an active treatment phase of up to 24 months during which patients were treated with teriparatide (which they could discontinue at any time); and (2) an ongoing post-treatment follow-up phase after the discontinuation of teriparatide treatment, which has a minimum duration of 18 months. In France and Sweden, the reimbursement of teriparatide was for 18 months only; in the other six countries, reimbursement was for 24 months. The primary objective of the ExFOS study is to determine the incidence of clinical vertebral and non-vertebral fractures in patients treated with teriparatide. The results presented in this paper are those from the planned interim analysis of patients who received active treatment for up to 24 months.

Physicians enrolled patients during routine clinical practice. Eligible patients were judged suitable for teriparatide treatment, were teriparatide-treatment naïve at enrolment and were prescribed teriparatide (20 μg administered once daily by subcutaneous self-injection) at the baseline visit. Patients were excluded if they were currently being treated with an investigational drug or procedure, or if they had contraindications to teriparatide [8]. In addition to, or after, treatment with teriparatide, patients could be treated with any pharmacological intervention prescribed by the physician for the treatment of osteoporosis. Patients gave written informed consent prior to enrolment and were able to withdraw without consequence at any time. The study was approved by local ethics committees or review boards, depending on local requirements.

### Data Collection and Assessments

All patient observations and data collection occurred within the normal course of clinical care. For the active treatment phase, data were collected at the baseline visit and at approximately 3, 6, 12, 18, and 24 months after starting teriparatide treatment, but physicians were not obliged to change their usual scheduling practice for the participating patients. For the analyses, actual patient visits were assigned to these times according to pre-defined time intervals.

Patient information recorded included demographics, medical history, comorbidities and concomitant medications, lifestyle and risk factors for osteoporosis and falls, BMD, osteoporotic fracture history (number and location), and previous and current osteoporosis therapies. Physicians recorded the date teriparatide treatment was started, whether reimbursement was provided for teriparatide treatment, and what type of reimbursement was provided (public or private health insurance). Patient adherence to teriparatide treatment was assessed at each visit by patient self-report.

#### Fracture Analysis

Patients were queried at each observation about the incidence of new clinical fractures, and the fracture location and date of fracture were recorded. A new or worsened clinical vertebral fracture was identified from the presence of a confirmed radiographic vertebral fracture associated with signs and/or symptoms suggestive of vertebral fracture as defined by Ross [15]. The radiographic definition of a new or worsened vertebral fracture was according to the physicians’ clinical practice. Distinction of low- versus high-trauma fractures was made according to the investigators’ assessment of the trauma force.

#### Health-Related Quality of Life

HRQoL was self-assessed by patients at each visit using the EuroQoL-5 Dimension (EQ-5D) questionnaire [16]. Patients rated their current health state in five domains (mobility, self-care, usual activities, pain/discomfort and anxiety/depression) scoring each domain on a 3-point scale. From the scores, a single Health State Value (EQ-5D HSV) was calculated based on the UK scoring algorithm [17]. In addition, patients rated their overall health status on the day of assessment using a visual analogue scale (VAS; EQ-VAS) ranging from 0 (worst imaginable health state) to 100 (best imaginable health state).

#### Back Pain

Back pain was self-assessed by patients at each visit using a back pain questionnaire [10] and a 100 mm VAS, which has been shown to be a reliable measure of pain [18]. Patients also indicated whether or not they had used analgesic medication for their back pain in the past month, and the type and frequency of analgesic medication used.

#### Safety

Spontaneously reported adverse events were collected throughout the study.

### Sample Size

A sample size of 1600 patients was calculated based on the primary outcome of the study (the incidence of clinical vertebral and non-vertebral fractures in patients treated with teriparatide) and using an estimated drop-out rate of 30 % and an estimated incident fracture rate at 24 months of 11 %, based on previous results from the EFOS study [10].

### Statistical Analysis

Data were analysed for the active treatment cohort, which included all patients with baseline data who received at least one dose of teriparatide and returned for a post-baseline visit. All models and analyses were pre-specified in a statistical analysis plan.

Baseline characteristics were summarised using descriptive statistics (frequency for categorical outcomes, and number, mean, standard deviation [SD] or median with interquartile ranges [Q1, Q3] for continuous variables).

Incident fractures, HRQoL and back pain were summarised over the teriparatide treatment period of up to 24 months. The number of fractures occurring in patients receiving teriparatide treatment was summarised in 6-month intervals and a logistic regression with repeated measures was used to assess the change in the proportion of patients with one or more fractures over time, as described previously for the EFOS study [10, 19]. The models for the odds of fracture were adjusted for visit and the following covariates: gender, age, prior bisphosphonate/denosumab use and a history of vertebral or non-vertebral fracture in the last 12 months before starting teriparatide. Contrasts were made between the odds of fracture in the first 6 months of treatment (0–6 months) and each subsequent 6-month period. The results are presented as odds ratios (OR), 95 % confidence intervals (95 % CI) and *p* values. The fracture analyses were repeated separately for clinical vertebral fractures, non-vertebral fractures and main non-vertebral fractures (fractures of the forearm/wrist, hip, humerus, leg and ribs).

A Cox proportional hazards model was used to evaluate the effect of baseline covariates on time to first on-study fracture. Any fractures occurring between treatment start and 24 months were included. The results are presented as hazard ratios (HR) and 95 % CI.

Changes from baseline in EQ-5D HSV, EQ-VAS score and back pain VAS score were analysed using mixed models for repeated measures (MMRM) adjusting for selected pre-specified variables which included age, duration of prior bisphosphonate therapy, number of fractures at baseline, fractures in the 12 months before starting teriparatide, and diagnosis of rheumatoid arthritis or other rheumatological disorder. The numbers of patients reporting an improvement, no change or worsening in back pain frequency, severity and limitations of activities during the last month were analysed using the Wilcoxon signed rank test to evaluate differences between the first visit period and the following periods.

All statistical analyses were performed using SAS version 9.3 (SAS institute Inc., Cary, USA).

## Results

### Patient Disposition and Characteristics

Of the 1611 patients enrolled in eight European countries, 1454 patients were included in the active treatment cohort. The remaining 157 patients did not have at least one post-baseline visit while receiving teriparatide. The disposition of patients in the active treatment cohort at each time point is shown in Online Resource 1. Of the 1454 patients included, there were 1240 reasons given for discontinuation of teriparatide in 1229 patients: treatment completed (*n* = 1018, 70.0 %), patient decision (*n* = 138, 9.5 %), physician decision (*n* = 41, 2.8 %), adverse event (*n* = 26, 1.8 %), death (*n* = 10, 0.7 %) and non-compliance (*n* = 7, 0.5 %). At the time of this interim database lock, teriparatide discontinuation or a reason for stopping teriparatide treatment was not recorded for 225 patients.

The baseline demographics and clinical characteristics of the active treatment cohort are summarised in Table 1 and were similar to the overall cohort [14]. The mean (SD) age of the active treatment cohort at baseline was 70.2 (9.8) years, all patients were Caucasian, 90.6 % were female, 14.4 % were taking glucocorticoids, 85.2 % had experienced previous fractures, 32.0 % had sustained a vertebral fracture in the 12 months before starting teriparatide and 88.7 % reported taking prior osteoporosis medication.

**Table 1:** Baseline characteristics of patients in the ExFOS active treatment cohort

Characteristic	Active treatment cohort (*N* = 1454)
Gender (females, males), *n* (%)	1318 (90.6), 136 (9.4)
Age (years), mean (SD)	70.2 (9.8)
Body mass index (kg/m^2^), mean (SD)	25.6 (4.5)
Patients with previous fracture, *n* (%)	1239 (85.2)
Number of previous fractures, median (Q1, Q3)	2.0 (1.0, 3.0)
Number of previous vertebral fractures, median (Q1, Q3)	2.0 (1.0, 3.0)
Patients with fractures in the 12 months before starting teriparatide, *n* (%)	688 (47.3)
Patients with vertebral fractures in the 12 months before starting teriparatide, *n* (%)	465 (32.0)
Patients with maternal history of hip fracture, *n* (%)	236 (19.5)
Uses arms when standing from chair, *n* (%)	756 (52.3)
Sight problems, *n* (%)	469 (32.5)
Current smoker, *n* (%)	214 (14.9)
Exercises ≥1 h/week, *n* (%)	861 (60.1)
Hours of exercise/week^a^, median (Q1, Q3)	4.0 (2.0, 7.0)
Has at least one alcoholic drink/week, *n* (%)	507 (35.7)
Number of patients with falls in previous year, *n* (%)	
1 fall	287 (20.5)
>1 fall	256 (18.2)
Immobilised for >12 months, *n* (%)	45 (3.1)
Reproductive history for females (*n* = 1318)^b^	
Reached menopause, *n* (%)	986 (98.5)
Years since onset of menopause, median (Q1, Q3)	23.0 (16.0, 29.0)
Early menopause (<40 years of age), *n* (%)	70 (5.8)
Surgical menopause, *n* (%)	137 (10.9)
Nulliparous, *n* (%)	147 (11.2)
Prior osteoporosis medication, *n* (%)	1289 (88.7)
Prior bisphosphonate use, *n* (%)	941 (64.7)
Duration of prior bisphosphonate therapy (months), mean (SD)	20.5 (36.6)
Current comorbidities, any disease, *n* (%)	487 (33.5)
Rheumatoid arthritis or other rheumatological disorder, *n* (%)	163 (11.2)
Taking glucocorticoids, *n* (%)	210 (14.4)

Percentages are based on patients with non-missing data

*SD* standard deviation, *Q1*, *Q3* first and third quartile of interquartile range

^a^For patients who reported exercising

^b^Fifteen women were premenopausal

### Osteoporosis Treatment

The median (Q1, Q3) and mean (SD) durations of teriparatide treatment were 23.7 (18.2, 24.0) months and 21.0 (4.8) months, respectively, for the 1303 patients who provided a teriparatide stop date by the time of this interim database lock. The stop date of teriparatide treatment was not available for 151 patients at the time of this interim database lock.

**Fig. 1:**
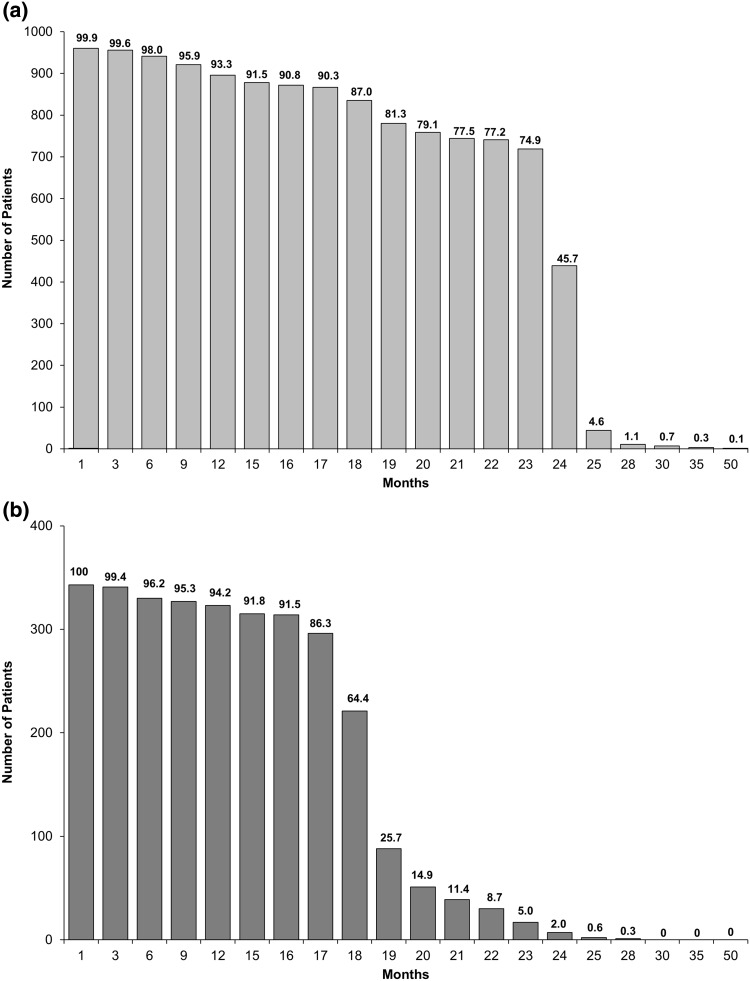
Number and percentage of patients still taking teriparatide after each month. **a** Countries with 24 months’ reimbursement for teriparatide (Croatia, Denmark, Greece, Italy, Norway, Slovenia). The number above each column is the percentage based on the number of patients with non-missing data (*n* = 960); data regarding teriparatide continuation missing for 77 patients. **b** Countries with 18 months’ reimbursement for teriparatide (France, Sweden). The number above each column is the percentage based on the number of patients with non-missing data (*n* = 343); data regarding teriparatide continuation missing for 74 patients

The number and percentage of patients still taking teriparatide after each month are shown in Fig. 1. The decrease in teriparatide use between 18 and 24 months occurred mostly in the two countries where teriparatide was reimbursed for 18 months (i.e. France and Sweden; *n* = 417 patients, 28.7 % of the active treatment cohort). The mean (SD) treatment duration for countries with 18 months’ reimbursement and 24 months’ reimbursement was 18.0 (3.6) months and 22.1 (4.8) months, respectively. During the 24-month period, 4.2 % of patients reported one or more treatment interruptions of longer than 4 weeks and the median (Q1, Q3) self-reported number of missed injections during the last month before each visit was 0 (0, 3).

After teriparatide was prescribed at the baseline visit, combination treatment with other osteoporosis medications was uncommon: 43 patients (3.0 %) were treated with bisphosphonates and 13 patients (0.9 %) with non-bisphosphonate antiresorptives at any time from baseline until the end of the active treatment phase. The majority of patients continued taking calcium and vitamin D supplementation (83.5 and 97 %, respectively) during teriparatide treatment.

### Fractures

Table 2 shows the incidence of clinical fractures during teriparatide treatment for the active treatment cohort regardless of the level of trauma. Of the 1454 patients, 103 (7.1 %) sustained a total of 122 clinical fractures between the start and end of active treatment. Of the 103 patients with fractures, 86 sustained a single fracture, 15 sustained two fractures and two patients sustained three fractures. Of the 122 fractures, 26 (21 %) were clinical vertebral fractures and 96 (79 %) were non-vertebral fractures; 68 (56 %) of all fractures were main non-vertebral fractures at the forearm/wrist (*n* = 31), hip (*n* = 12), humerus (*n* = 9), leg (*n* = 9) or ribs (*n* = 7). Table 2 shows that there was a 45 and 49 % decrease in the odds of clinical fractures in the >12- to 18-month and >18- to 24-month periods versus the first 6-month period, respectively (*p* < 0.05).

**Table 2:** Clinical fractures during teriparatide treatment (0–24 months) for the active treatment cohort

Time interval (months)	*N* ^a^	Number of fractures per 10,000 patient years	Total number of fractures	Patients with ≥1 fracture, *n* (%)^b^	Odds of fracture (95 % CI)^c^	Odds ratio^c,d^ (95 % CI)	*p* value^d^
0–6	1454	670	48	44 (3.0 %)	0.017 (0.009–0.031)	–	–
>6–12	1384	461	31	28 (2.0 %)	0.011 (0.006–0.021)	0.66 (0.42–1.04)	0.075
>12–18	1295	401	25	22 (1.7 %)	0.009 (0.005–0.019)	0.55 (0.33–0.92)	0.022
>18–24	1087	436	18	17 (1.6 %)	0.009 (0.004–0.018)	0.51 (0.29–0.90)	0.021
Total^e^	1454		122	103 (7.1 %)			

*CI* confidence interval

^a^ *N* = all patients with information regarding fractures within the time window

^b^As some patients experienced a fracture in more than one time interval, the total was not the sum of patients with a fracture in each interval

^c^Adjusted model by gender, age, prior bisphosphonate/denosumab use, and history of vertebral or non-vertebral fracture in the 12 months before starting teriparatide

^d^Compared with 0- to 6-month interval

^e^All fractures from treatment start to end of treatment within the 24 months are included

**Fig. 2:**
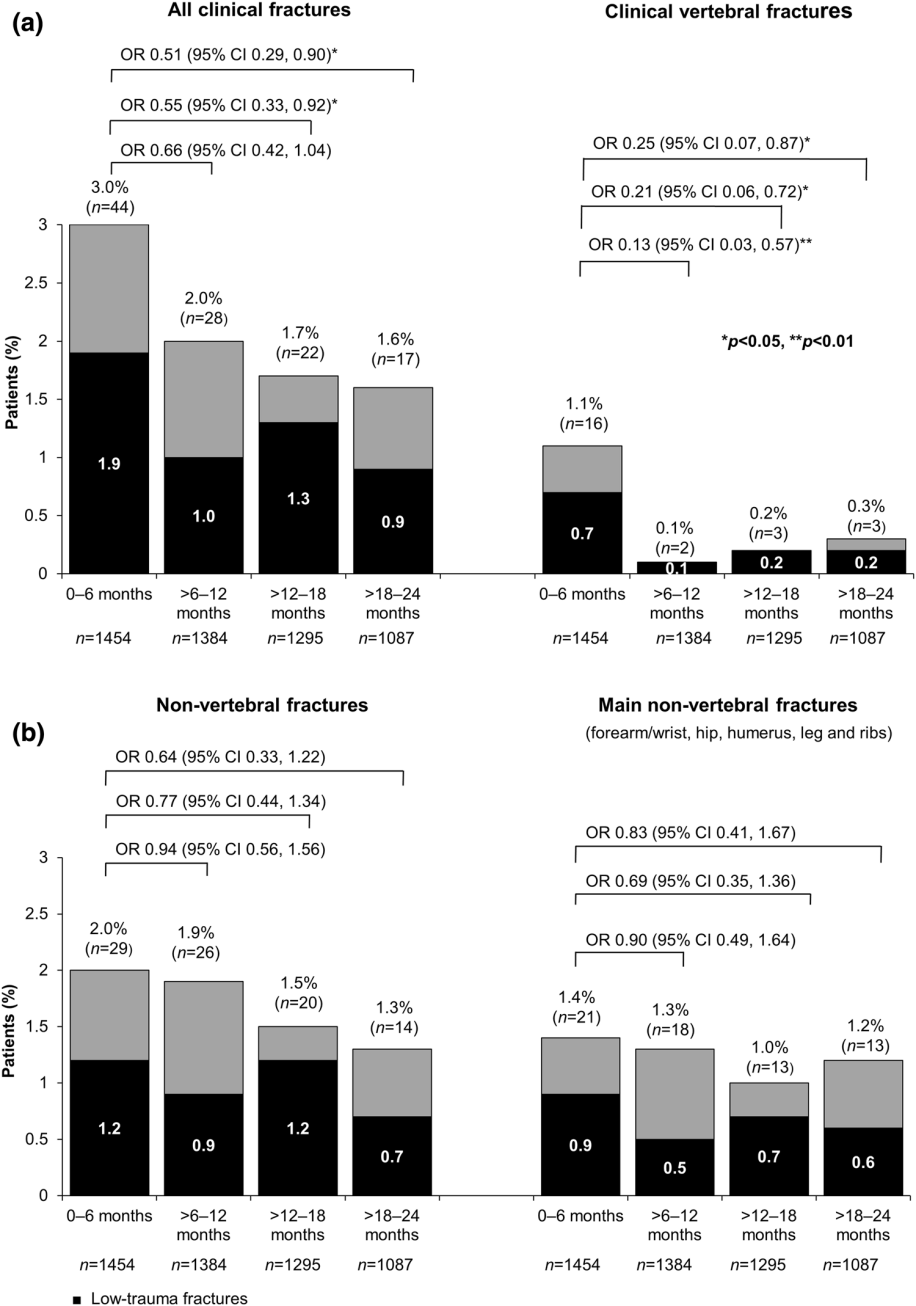
Patients with fractures in each 6-month interval by fracture type. The top of each column gives the *n* (%) of patients with ≥1 fracture. The OR and 95 % CI compare the numbers of patients with ≥1 clinical fracture (regardless of the level of trauma) in 6-month intervals against the first 6 months. *p < 0.05, **p < 0.01. *Note* as some patients experienced more than one type of fracture, the number of patients for all clinical fractures is not the sum of patients with clinical vertebral fractures and non-clinical vertebral fractures. Also, as some patients experienced a fracture in more than one time interval, the total was not the sum of patients with a fracture in each interval

Over the 24-month period, 67 patients (4.6 %) sustained a total of 77 low-trauma clinical fractures. Figure 2 presents the number (%) of patients with all clinical fractures and clinical vertebral, non-vertebral and main non-vertebral fractures in each 6-month period (also see Online Resource 2). For clinical vertebral fractures, there was a significant reduction in the adjusted odds of fracture during each of the teriparatide treatment periods (87 % decrease in the >6- to 12-month period, 79 % decrease in the >12- to 18-month period and 75 % decrease in the >18- to 24-month period) compared with the first 6 months of teriparatide treatment. The adjusted odds of non-vertebral fractures and main non-vertebral fractures during >6–24 months of teriparatide treatment did not differ significantly from those in the first 6 months (Fig. 2 and Online Resource 2).

The relative fracture risk was greater [HR = 1.59 (95 % CI 1.07 to 2.36)] for patients with a fracture versus those without a fracture in the 12 months before starting teriparatide. In addition, the relative risk of fracture was greater [HR = 3.60 (95 % CI 1.14 to 11.42)] for female versus male patients.

### HRQoL

**Fig. 3:**
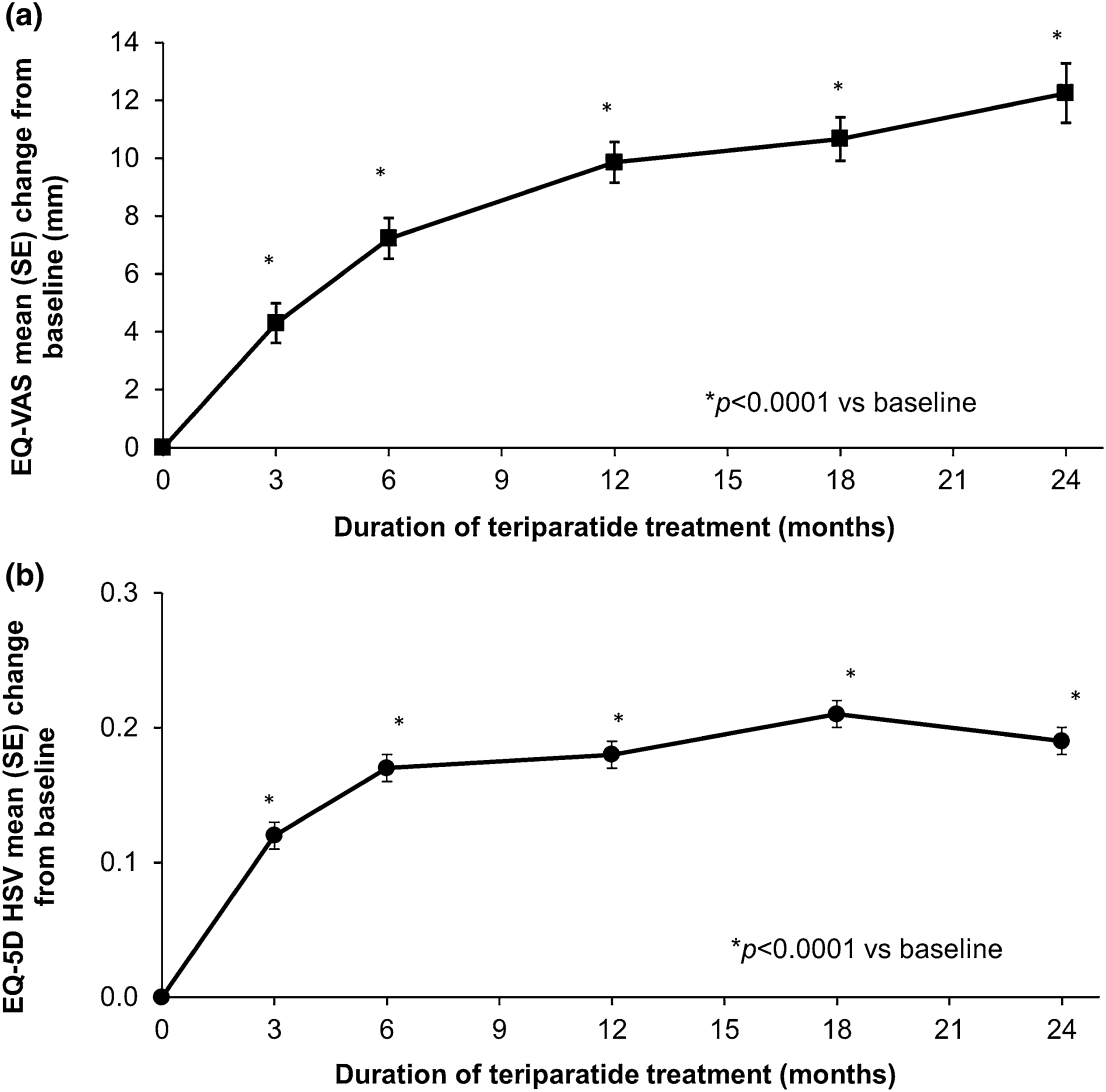
HRQoL: **a** EQ-VAS and **b** EQ-5D HSV adjusted least square mean (SE) change from baseline. Data presented are from MMRM analyses. Models included change from baseline in EQ-VAS or EQ-5D HSV as dependent variable, visit as a fixed repeated effect, and baseline score (EQ-VAS or EQ-5D HSV), age, duration of prior bisphosphonate therapy, number of previous fractures, fractures in the 12 months before starting teriparatide and diagnosis of rheumatoid arthritis or other rheumatological disorder as covariates. *p < 0.0001 compared with baseline. The mean (SD) EQ-VAS values at baseline 3, 6, 12, 18 and 24 months were 56.6 (21.2), 61.8 (19.6), 64.8 (19.9), 67.8 (19.1), 69.4 (19.1) and 72.3 (19.7), respectively. The mean (SD) EQ-5D HSV scores at baseline 3, 6, 12, 18 and 24 months were 0.50 (0.36), 0.63 (0.30), 0.68 (0.27), 0.70 (0.27), 0.74 (0.25) and 0.76 (0.24), respectively

The mean (SD) EQ-VAS score at baseline was 56.6 (21.2) and the mean (SD) EQ-5D HSV at baseline was 0.50 (0.36) with a median (Q1, Q3) of 0.62 (0.19, 0.76). The adjusted mean changes in EQ-5D HSV and EQ-VAS score from baseline during teriparatide treatment (Fig. 3) show significant improvements at all post-baseline time points. In the MMRM for EQ-VAS, several covariates had a potential influence on the change in HRQoL: there was less improvement if the baseline EQ-VAS score was higher (−0.64 for each additional 1 mm; *p* < 0.0001), and if the patient was older (−0.10 for each additional year; *p* < 0.010) or had more previous fractures (−0.82 for each additional fracture; *p* = 0.0002), a diagnosis of rheumatoid arthritis or other rheumatological disorder (−2.64 compared with no such diagnosis; *p* = 0.033) and fractures in the 12 months before starting teriparatide (−2.80 compared with no fractures; *p* = 0.0006). Similarly, in the MMRM for EQ-5D HSV, there was less improvement if the patient had a lower baseline EQ-5D HSV (−0.68 for each 1 point; 95 % CI −0.72 to −0.65; *p* < 0.0001), more previous fractures (−0.02 for each additional fracture; 95 % CI −0.02 to −0.01; *p* < 0.0001) and a diagnosis of rheumatoid arthritis or other rheumatological disorder (−0.04 compared with no such diagnosis; 95 % CI −0.07 to 0.00; *p* = 0.041).

The EQ-5D domain changes over time showing the greatest improvements were reported in the pain/discomfort and usual activities domains (Online Resource 3).

### Back Pain

**Fig. 4:**
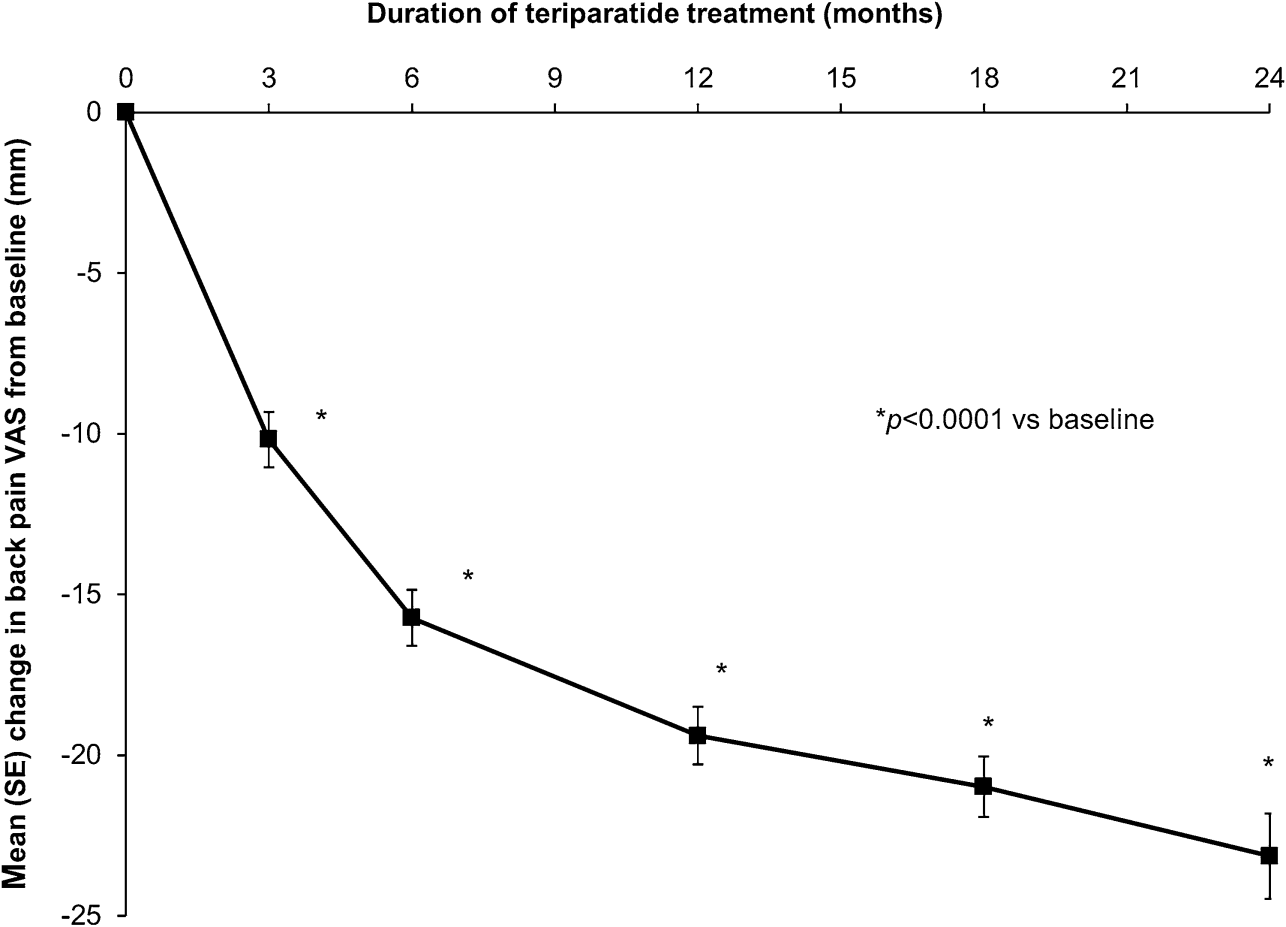
Back pain VAS: adjusted least square mean (SE) change from baseline. Data presented are from MMRM analysis. Model included change from baseline in back pain VAS as dependent variable, visit as a fixed repeated effect and baseline back pain VAS, age, duration of prior bisphosphonate therapy, number of vertebral fractures at baseline, vertebral fractures in the 12 months before starting teriparatide treatment and diagnosis of rheumatoid arthritis or other rheumatological disorder as covariates. *p < 0.0001 compared with baseline. The mean (SD) back pain VAS scores at baseline 3, 6, 12, 18 and 24 months were 50.1 (27.0), 41.0 (25.3), 35.4 (24.3), 31.6 (23.6), 29.7 (23.9), and 27.2 (23.5) mm, respectively

The mean (SD) back pain VAS score at baseline was 50.1 (27.0) mm. Figure 4 shows a decrease (i.e. improvement) in the adjusted mean change in back pain VAS from baseline over time during up to 24 months of treatment with teriparatide; the decrease in pain was significant at all post-baseline assessment points, starting at 3 months. Of the variables included in the MMRM, two had a significant effect on the change in back pain VAS: there was a bigger improvement (i.e. decrease) if the back pain VAS was higher at baseline (−0.58 mm for each additional 1 mm; 95 % CI −0.62 to −0.55; *p* < 0.0001); there was less improvement if the patient had more previous fractures (1.64 mm for each additional fracture; 95 % CI 1.04–2.24; *p* < 0.0001).

The results from the back pain questionnaire showed that the frequency and severity of back pain and limitations of activities and days in bed due to back pain decreased during teriparatide treatment for up to 24 months (Online Resource 4). At every post-baseline visit, significantly more patients reported a decrease from baseline in the frequency of back pain, severity of back pain and limitations of activities, compared with an increase from baseline in these measures (all *p* < 0.001). The majority of patients (75.2 %) reported taking analgesic medication for back pain in the month before the baseline assessment. The proportions of patients reporting analgesic use decreased to 63.5 and 58.6 % at 18 and 24 months of teriparatide treatment, respectively. Paracetamol was the most commonly used analgesic medication at all time points, followed by acetylsalicylic acid/non-steroidal anti-inflammatory drugs, and then low-potency and high-potency opiates (Online Resource 4).

### Safety

Of the 1611 enrolled patients, 173 (10.7 %) had at least one adverse event and 120 (7.4 %) had at least one serious adverse event during the active treatment phase. Of the 339 adverse events reported, 211 (62.2 %) were serious and 57 (16.8 %) were considered possibly related to study medication. The most common adverse events (>2 %) reported were fall (7.1 %), nausea (4.1 %) and headache (2.9 %). No cases of osteosarcoma were observed during the 24-month teriparatide treatment phase. There were 34 patients with at least one adverse event leading to death (2.1 % of all 1611 enrolled patients); none of these deaths were considered related to the study drug by the reporting investigators.

## Discussion

This analysis of the active treatment phase of the ExFOS study confirms the effectiveness of teriparatide treatment for up to 24 months (an extra 6 months versus the originally approved 18-month treatment period in Europe) in reducing the incidence of clinical fractures, improving HRQoL and reducing back pain in patients with severe osteoporosis. The risk for any clinical fracture after >18–24 months of teriparatide treatment was reduced by 49 % compared with the first 6-month period of treatment. Notably, the risk for clinical vertebral fractures was low and was significantly reduced by 87 % at >6–12 months, 79 % at >12–18 months and 75 % at >18–24 months, compared with the first 6 months of treatment. This reduction in clinical fractures was accompanied by an improvement in HRQoL and a reduction in back pain at all post-baseline assessments during teriparatide treatment for up to 24 months.

Our results are consistent with those of other observational studies of teriparatide use including the EFOS study, which was conducted only in postmenopausal women with severe osteoporosis who were treated with teriparatide for up to 18 months (the approved duration for treatment in the EU before 2009) [10]. The ExFOS study extends these findings to other European countries (Croatia, Italy, Norway and Slovenia), includes a broader range of patients with osteoporosis [i.e. men and premenopausal women with glucocorticoid-induced osteoporosis (GIO)] and allowed a longer duration of teriparatide treatment of up to 24 months. However, the incidence of fractures per 10,000 patient years was lower in ExFOS (401 at 12–18 months, 436 at 18–24 months; Table 2) than in EFOS (583 at 12–18 months) [10], probably indicating a less severely osteoporotic patient population at baseline, with a lower risk for fracture. Also, the ExFOS study included men (*n* = 136) and premenopausal women with GIO (*n* = 15), who have a lower risk of fractures than postmenopausal women. The fracture baseline characteristics of the EFOS population, reported by Rajzbaum et al. [20], would also support the hypothesis that the ExFOS population had less severe osteoporosis since the reimbursement criteria for teriparatide were less strict in some of the participant countries in the later study.

A recent observational study in patients with severe osteoporosis referred to a specialist clinic in Scotland found that patients treated with teriparatide (for either 18 or 24 months) had a greater increase in lumbar spine BMD (8.2 vs. 5.0 % per year, *p* = 0.018) and a lower incidence of clinical vertebral fractures (1.4 vs. 6.6 %, *p* = 0.011) compared with patients receiving standard care (primarily oral bisphosphonate therapy) [21]. Consistent with our results, teriparatide treatment was associated with a reduced risk of clinical vertebral fracture (about 88 %), although the timescale of follow-up for teriparatide recipients was longer (37.4 months) [21].

The fracture results from ExFOS can also be compared with those from the DANCE observational study in the USA, which used a similar analysis to evaluate the incidence of new non-vertebral fragility fractures in patients treated with teriparatide for up to 24 months [13]. In DANCE, the incidence of new non-vertebral fractures was significantly reduced during teriparatide treatment (to 0.91, 0.70 and 0.81 % for the >6–12 months’, >12–18 months’ and >18–24 months’ treatment periods, respectively) compared with the first 6-month period (1.42 %) [13]. In ExFOS, as shown in Fig. 2, the incidence of non-vertebral fractures was also numerically reduced during teriparatide treatment (from 2.0 % for 0–6 months, to 1.9, 1.5 and 1.3 % for >6–12, >12–18 and >18–24 months, respectively), but the odds of a non-vertebral fracture during the later time periods did not differ significantly from that in the first 6-month period. Possible reasons for the differential findings are that the ExFOS patient cohort was much smaller (1454 vs. 3720 patients in DANCE [13]) and that there were differences in baseline characteristics between the two study populations; for example, as seen in Table 1, fewer patients in ExFOS had comorbid conditions (33.5 vs. 83.1 % patients in DANCE) [13].

Observational studies reflect routine clinical practice and provide useful information on a larger and more diverse group of real-world patients than those carefully selected to participate in RCTs [22]. This includes estimates of compliance and persistence with osteoporosis therapy, which can impact on outcomes such as fracture rates and healthcare resource use. Persistence is usually defined as the time to treatment discontinuation or the proportion of patients that fill a prescription without a treatment gap of 30, 60 or 90 days [1]. In a meta-analysis of observational studies, non-persistence with osteoporosis therapy increased the fracture risk by 30–40 % [23]. A recent, large-scale retrospective analysis of a US claims database showed that women noncompliant with their osteoporosis treatment had a higher risk of fractures and incurred higher medical costs than compliant patients [24].

As teriparatide is self-administered once daily by subcutaneous injection, compliance and/or persistence may differ from osteoporosis medications that are taken orally or injected less frequently. Moreover, teriparatide persistence has not been well-characterised beyond 12 months among patients with severe osteoporosis and may decline over longer periods of treatment. Analyses of claims databases have shown an inverse relationship between persistence with teriparatide therapy over 24 months and fracture risk [25, 26]. Nevertheless, treatment effectively reduces fractures after only a few months and, thereafter, persistent patients show the lowest fracture incidence [26, 27].

More than 90 % of patients in our study were still taking teriparatide after 12 months of treatment and a high level of persistence was maintained during continued teriparatide treatment: 86 % at 17 months for countries with 18-month reimbursement and 75 % at 23 months for countries with 24-month reimbursement (Fig. 1). The decline in persistence between 23 and 24 months is most likely because, in some countries with 24-month reimbursement for teriparatide, reimbursement only covers 24 pen devices, which corresponds to 22.4 months of treatment. The low frequency of self-reported treatment interruptions longer than 4 weeks and of missed injections also suggests good treatment compliance. Overall, these results suggest that the majority of patients persist with teriparatide treatment when it is prescribed for up to 24 months.

Also, the majority of patients being prescribed teriparatide in Europe have very severe osteoporosis with high levels of back pain and disability after prior treatment with other antiresorptive osteoporosis medications. The early and sustained improvements in HRQoL and back pain we observed may have had a positive effect in reinforcing persistence with teriparatide. Moreover, the participant countries in the study have patient support programmes in place and, in most cases, patients are followed in specialised osteoporosis units that may facilitate adherence and persistence to injectable therapies such as teriparatide [28–30]. Alternatively, policies exist that involve the close monitoring of patients at specialised osteoporosis units.

Consistent with the HRQoL results from EFOS [10, 31], patients in ExFOS had low baseline scores (EQ-5D HSV and EQ-VAS) that improved significantly during teriparatide treatment for up to 24 months. Notably, the steady improvement in HRQoL was maintained during the additional 6 months of teriparatide therapy. The changes in EQ-5D (HSV and EQ-VAS) remained significant after adjustment for various factors including age, baseline score, previous fractures, prior bisphosphonate use and diagnosis of rheumatoid arthritis. Improvements were seen across all five EQ-5D domains but were greatest in the domains of usual activities and pain/discomfort. Improvements in HRQoL were smaller in older patients and in patients with recent previous fractures or a diagnosis of rheumatoid arthritis. This is in contrast with EFOS, where Ljunggren et al. [31] found that the improvement in HRQoL during teriparatide treatment was lower in the subgroups with incident clinical fractures but was unaffected by previous fracture in the 12 months before starting teriparatide.

Back pain is common in patients with osteoporosis and has been linked to the number and severity of vertebral fractures [3, 32]. The patients taking part in ExFOS had a high back pain VAS score at baseline (mean 50.1 mm), reflecting the severity of their osteoporosis and the high prevalence of vertebral fractures in the 12 months before starting teriparatide (32 %). Nevertheless, we observed a marked reduction in back pain VAS during teriparatide treatment for up to 24 months. A decrease of 10 mm in back pain VAS is considered a clinically significant reduction [33], and this was exceeded by the 3-month observation in ExFOS; Fig. 4 shows the decrease in back pain VAS had reached 23 mm by 24 months. As seen in Fig. 4, there was a continued reduction in back pain from baseline during the extra 6 months of teriparatide therapy (months 18–24). This was accompanied by reductions in the frequency and severity of back pain, and activity limitations and days in bed due to back pain, together with self-reported reductions in the use of analgesics for back pain. An earlier study [34] and a meta-analysis [35] found a reduced incidence of new or worsening back pain in teriparatide-treated patients, which may have been associated with a reduction in vertebral fractures [34]. Among postmenopausal women with severe osteoporosis who received teriparatide for 2 years in the EUROFORS study, there was a rapid and significant reduction in back pain in both subgroups of patients with and without a vertebral fracture in the 12 months before baseline, despite the high pain level (mean baseline back pain VAS was 54.8 mm with vs. 45.8 mm without a recent vertebral fracture) [36].

The adverse events spontaneously reported during teriparatide treatment were consistent with the current label information. Notably, there were no cases of osteosarcoma, a rare bone cancer found previously in preclinical studies of rats exposed to teriparatide for 2 years [37]. This is in agreement with the findings from post-marketing surveillance studies in the USA and five Nordic countries, which failed to identify a causal association between teriparatide treatment and osteosarcoma in humans [38, 39]. Moreover, a retrospective longitudinal cohort study using Danish nationwide registers, which included 4104 subjects, reported that osteosarcoma has not been diagnosed in any Danish patient receiving teriparatide since it was introduced on the market in 2003 [40].

Several study limitations have to be taken into account when interpreting the current findings. Notably, this was an observational study in a naturalistic setting and the data collected by patient self-report (including fracture data) may be subject to recall bias. Also, the duration of teriparatide therapy varied as a consequence of the reimbursement criteria for different countries: teriparatide was only reimbursed for 18 months in France and Sweden (*n* = 417 patients; 28.7 % of the active treatment cohort) and 24 months in the other participating countries. Finally, patients were not evenly distributed across the participating countries and previous research has shown that there is a marked difference in the incidence of fractures between countries [1].

The strengths of the ExFOS study include the observational study design that comprised a broad population of patients with severe osteoporosis according to the approved European label. This allowed us to gather longitudinal data in patients treated with teriparatide in real-life clinical practice, making the results applicable to the general population in Europe. Also, our pre-defined analyses adjusted for factors that might influence the risk for fracture, including age, duration of prior bisphosphonate therapy, previous fractures and comorbid rheumatological disorders.

In conclusion, this analysis of the active treatment phase of the ExFOS study shows that men and women with severe osteoporosis treated with teriparatide in routine clinical practice experience a significant reduction in incident fracture rate over 18–24 months of teriparatide treatment. This is accompanied by a significant improvement in HRQoL and a significant reduction in back pain. Safety was consistent with the current prescribing information for teriparatide. Although our findings should be interpreted in the context of the open-label, non-controlled design of the study, they indicate that teriparatide is an effective treatment for patients with osteoporosis for up to 24 months in routine clinical practice. Further results from the post-teriparatide follow-up period of the study will be reported at a later date.

## Electronic supplementary material

Below is the link to the electronic supplementary material.

Supplementary material 1 (TIFF 898 kb)

Supplementary material 2 (PDF 202 kb)

Supplementary material 3 (PDF 100 kb)

Supplementary material 4 (PDF 106 kb)
