# Supplementary figures and images for: Fracture Rate, Quality of Life and Back Pain in Patients with Osteoporosis Treated with Teriparatide: 24-Month Results from the Extended Forsteo Observational Study (ExFOS)
Source: Calcif Tissue Int. 2016 Apr 30;99:259–71. doi: 10.1007/s00223-016-0143-5 (PMC4960288; doi:10.1007/s00223-016-0143-5)

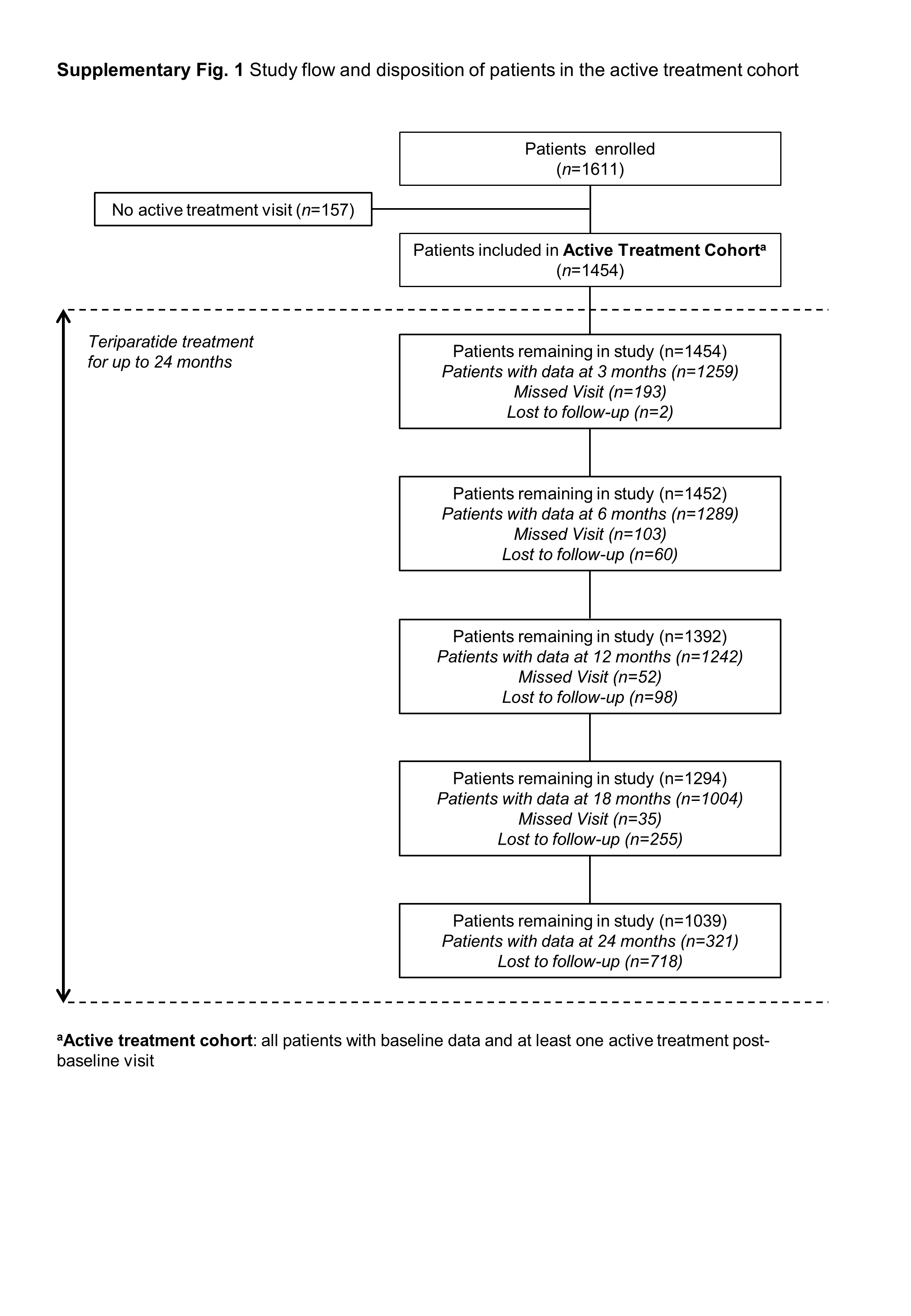

Supplement: Supplementary file 1 — Supplementary material 1 (TIFF 898 kb) [file 223_2016_143_MOESM1_ESM.tif]
