# Supplementary material for: Fracture Rate, Quality of Life and Back Pain in Patients with Osteoporosis Treated with Teriparatide: 24-Month Results from the Extended Forsteo Observational Study (ExFOS)
Source: Calcif Tissue Int. 2016 Apr 30;99:259–71. doi: 10.1007/s00223-016-0143-5 (PMC4960288; doi:10.1007/s00223-016-0143-5)
Supplement: Supplementary file 2 — Supplementary material 2 (PDF 202 kb) [file 223_2016_143_MOESM2_ESM.pdf]

Online Resource 2: Clinical vertebral and non-vertebral fractures during teriparatide treatment (0 to 24 months) for the active treatment cohort

**Time interval (months)**	***N*[a]**	**Number of fractures per 10,000 patient years**	**Total number of fractures**	**Patients with ≥1 fracture, *n* (%)[b]**	**Odds of fracture (95% CI)[c]**	**Odds ratio[c,d] (95% CI)**	***p* value[d]**
Clinical vertebral fractures							
0 to 6	1454	237	17	16 (1.1)	0.006 (0.002–0.017)	-	-
>6 to 12	1384	45	3	2 (0.1)	0.001 (0.000–0.004)	0.13 (0.03–0.57)	0.007
>12 to 18	1295	48	3	3 (0.2)	0.001 (0.000–0.005)	0.21 (0.06–0.72)	0.013
>18 to 24	1087	73	3	3 (0.3)	0.001 (0.000–0.006)	0.25 (0.07–0.87)	0.029
Total[e]	1454		26	24 (1.7)			
Non-vertebral fractures							
0 to 6	1454	433	31	29 (2.0)	0.010 (0.005–0.021)	-	-
>6 to 12	1384	416	28	26 (1.9)	0.009 (0.004–0.020)	0.94 (0.56–1.56)	0.800
>12 to 18	1295	353	22	20 (1.5)	0.008 (0.003–0.018)	0.77 (0.44–1.34)	0.348
>18 to 24	1087	363	15	14 (1.3)	0.006 (0.003–0.016)	0.64 (0.33–1.22)	0.177
Total[e]	1454		96	82 (5.6)			
Main non-vertebral fractures (forearm/wrist, hip, humerus, leg and ribs)							
0 to 6	1454	321	23	21 (1.4)	0.006 (0.002–0.018)	-	-
>6 to 12	1384	283	19	18 (1.3)	0.006 (0.002–0.016)	0.90 (0.49–1.64)	0.721
>12 to 18	1295	209	13	13 (1.0)	0.004 (0.002–0.013)	0.69 (0.35–1.36)	0.283
>18 to 24	1087	315	13	13 (1.2)	0.005 (0.002–0.018)	0.83 (0.41–1.67)	0.601
Total[e]	1454		68	61 (4.2)			

[a]*N* = all patients with information regarding fractures within the time window

[b]As some patients experienced a fracture in more than one time interval, the total was not the sum of patients with a fracture in each interval

[c]Adjusted model by gender, age, prior bisphosphonate/denosumab use, and history of vertebral or non-vertebral fracture in the 12 months before starting teriparatide.

[d]Compared with 0- to 6-month interval

[e]All fractures from treatment start to end of treatment within the 24 months are included

CI, confidence interval
