# Supplementary material for: Fracture Rate, Quality of Life and Back Pain in Patients with Osteoporosis Treated with Teriparatide: 24-Month Results from the Extended Forsteo Observational Study (ExFOS)
Source: Calcif Tissue Int. 2016 Apr 30;99:259–71. doi: 10.1007/s00223-016-0143-5 (PMC4960288; doi:10.1007/s00223-016-0143-5)
Supplement: Supplementary file 3 — Supplementary material 3 (PDF 100 kb) [file 223_2016_143_MOESM3_ESM.pdf]

Online Resource 3: HRQoL: EQ-5D domains

	**Baseline (*N* = 1454)**	**3 months (*N* = 1259)**	**6 months (*N* = 1289)**	**12 months (*N* = 1242)**	**18 months (*N* = 1004)**	**24 months (*N* = 321)**
**Mobility**	(*n* = 1409)[a]	(*n* = 1178)	(*n* = 1191)	(*n* = 1127)	(*n* = 899)	(*n* = 259)
No problem, *n* (%)	568 (40.3)	590 (50.1)	659 (55.3)	671 (59.5)	595 (66.2)	167 (64.5)
Some problem, *n* (%)	792 (56.2)	568 (48.2)	525 (44.1)	451 (40.0)	302 (33.6)	91 (35.1)
Extreme problem, *n* (%)	49 (3.5)	20 (1.7)	7 (0.6)	5 (0.4)	2 (0.2)	1 (0.4)
**Self-care**	(*n* = 1405)	(*n* = 1175)	(*n* = 1190)	(*n* = 1126)	(*n* = 897)	(*n* = 258)
No problem, *n* (%)	910 (64.8)	856 (72.9)	933 (78.4)	910 (80.8)	743 (82.8)	216 (83.7)
Some problem, *n* (%)	434 (30.9)	294 (25.0)	235 (19.7)	203 (18.0)	143 (15.9)	38 (14.7)
Extreme problem, *n* (%)	61 (4.3)	25 (2.1)	22 (1.8)	13 (1.2)	11 (1.2)	4 (1.6)
**Usual activity**	(*n* = 1406)	(*n* = 1179)	(*n* = 1193)	(*n* = 1130)	(*n* = 899)	(*n* = 259)
No problem, *n* (%)	433 (30.8)	544 (46.1)	578 (48.4)	629 (55.7)	528 (58.7)	144 (55.6)
Some problem, *n* (%)	784 (55.8)	561 (47.6)	548 (45.9)	454 (40.2)	336 (37.4)	97 (37.5)
Extreme problem, *n* (%)	189 (13.4)	74 (6.3)	67 (5.6)	47 (4.2)	35 (3.9)	18 (6.9)
**Pain and discomfort**	(*n* = 1410)	(*n* = 1179)	(*n* = 1193)	(*n* = 1126)	(*n* = 899)	(*n* = 259)
None, *n* (%)	178 (12.6)	257 (21.8)	349 (29.3)	386 (34.3)	374 (41.6)	119 (45.9)
Moderate, *n* (%)	938 (66.5)	804 (68.2)	772 (64.7)	682 (60.6)	492 (54.7)	134 (51.7)
Extreme, *n* (%)	294 (20.9)	118 (10.0)	72 (6.0)	58 (5.2)	33 (3.7)	6 (2.3)
**Anxiety and depression**	(*n* = 1406)	(*n* = 1176)	(*n* = 1189)	(*n* = 1124)	(*n* = 898)	(*n* = 258)
None, *n* (%)	606 (43.1)	610 (51.9)	664 (55.8)	642 (57.1)	550 (61.2)	176 (68.2)
Moderate, *n* (%)	667 (47.4)	496 (42.2)	471 (39.6)	430 (38.3)	314 (35.0)	78 (30.2)
Extreme, *n* (%)	133 (9.5)	70 (6.0)	54 (4.5)	52 (4.6)	34 (3.8)	4 (1.6)

[a]Number of patients with non-missing entries (denominator for percentages) for all domains at all time points
