# Supplementary material for: Fracture Rate, Quality of Life and Back Pain in Patients with Osteoporosis Treated with Teriparatide: 24-Month Results from the Extended Forsteo Observational Study (ExFOS)
Source: Calcif Tissue Int. 2016 Apr 30;99:259–71. doi: 10.1007/s00223-016-0143-5 (PMC4960288; doi:10.1007/s00223-016-0143-5)
Supplement: Supplementary file 4 — Supplementary material 4 (PDF 106 kb) [file 223_2016_143_MOESM4_ESM.pdf]

Online Resource 4: Frequency and severity of back pain, limitations of daily activities, days in bed due to back pain, and analgesic use in the last month

	**Baseline (*N*= 1454)**	**3 months (*N* = 1259)**	**6 months (*N* = 1289)**	**12 months (*N* = 1242)**	**18 months (*N* = 1004)**	**24 months (*N*= 321)**
**Back pain experienced**	(*n* = 1444)[a]	(*n* = 1256)	(*n* = 1284)	(*n* = 1234)	(*n* = 1000)	(*n* = 312)
Yes, *n* (%)	1311 (90.8)	1095 (87.2)	1075 (83.7)	984 (79.7)	740 (74.0)	219 (70.2)
No, *n* (%)	133 (9.2)	161 (12.8)	209 (16.3)	250 (20.3)	260 (26.0)	93 (29.8)
**Frequency of back pain**	(*n* = 1311)	(*n* = 1095)	(*n* = 1075)	(*n* = 984)	(*n* = 740)	(*n* = 219)
Once or twice, *n* (%)	120 (9.2)	214 (19.5)	293 (27.3)	302 (30.7)	273 (36.9)	81 (37.0)
A few times, *n* (%)	180 (13.7)	275 (25.1)	258 (24.0)	224 (22.8)	151 (20.4)	46 (21.0)
Fairly often, *n* (%)	217 (16.6)	208 (19.0)	171 (15.9)	167 (17.0)	111 (15.0)	29 (13.2)
Every/almost every day, *n* (%)	794 (60.0)	398 (36.3)	353 (32.8)	291 (29.6)	205 (27.7)	63 (28.8)
**Severity of back pain**	(*n* = 1358)	(*n* = 1146)	(*n* = 1122)	(*n* = 1024)	(*n* = 757)	(*n* = 220)
Minor, *n* (%)	257 (18.9)	443 (38.7)	525 (46.8)	544 (53.1)	414 (54.7)	125 (56.8)
Moderate/severe, *n* (%)	1101 (81.1)	703 (61.3)	597 (53.2)	480 (46.9)	343 (45.3)	95 (43.1)
**Limitation of activities**[b]	(*n* = 1375)	(*n* = 1166)	(*n* = 1158)	(*n* = 1060)	(*n* = 809)	(*n* = 238)
None, *n* (%)	265 (19.3)	394 (33.8)	426 (36.8)	429 (40.5)	348 (43.0)	93 (39.1)
Minor, *n* (%)	278 (20.2)	328 (28.1)	351 (30.3)	317 (29.9)	246 (30.4)	79 (33.2)
Moderate/severe, *n* (%)	832 (60.5)	444 (38.1)	381 (32.9)	314 (29.6)	215 (26.6)	66 (27.7)
**Days in bed**[a]	(*n* = 1387)	(*n* = 1183)	(*n* = 1178)	(*n* = 1101)	(*n* = 845)	(*n* = 254)
None, *n* (%)	1140 (82.2)	1069 (90.4)	1106 (93.9)	1036 (94.1)	802 (94.9)	240 (94.5)
At least one, *n* (%)	247 (17.8)	114 (9.6)	72 (6.1)	65 (5.9)	43 (5.1)	14 (5.5)
Median (Q1, Q3)[c]	8 (3, 15)	5 (2, 14)	5 (2, 6.5)	3 (1, 5)	2 (1, 5)	3.5 (2, 5)
**Any analgesic use**	(*n* = 1387)	(*n* = 1171)	(*n* = 1156)	(*n* = 1047)	(*n* = 768)	(n = 220)
Yes, *n* (%)	1043 (75.2)	764 (65.2)	703 (60.8)	647 (61.8)	488 (63.5)	129 (58.6)
No, *n* (%)	344 (24.8)	407 (34.8)	453 (39.2)	400 (38.2)	280 (36.5)	91 (41.4)
**Type of analgesic**	(*n* = 1043)	(*n* = 764)	(*n* = 703)	(*n* = 647)	(*n* = 488)	(*n* = 129)
Paracetamol, *n* (%)	803 (77.0)	572 (74.9)	528 (75.1)	494 (76.4)	355 (72.7)	92 (71.3)
ASA/NSAIDs, *n* (%)	358 (34.3)	204 (26.7)	173 (24.6)	158 (24.4)	110 (22.5)	32 (24.8)
Low-potency opiates, *n* (%)	254 (24.4)	169 (22.1)	153 (21.8)	139 (21.5)	96 (19.7)	26 (20.2)
High-potency opiates, *n* (%)	118 (11.3)	62 (8.1)	68 (9.7)	62 (9.6)	42 (8.6)	11 (8.5)

[a]Number of patients with non-missing entries (denominator for percentages) for all back pain variables and analgesic use at all time points

[b]Due to back pain

[c]For those patients with at least one day in bed due to back pain

ASA, acetylsalicylic acid; NSAIDs, non-steroidal anti-inflammatory drugs
